# Supplementary material for: Comparison of CRISPR-Cas13b RNA base editing approaches for USH2A-associated inherited retinal degeneration
Source: Commun Biol. 2025 Feb 8;8:200. doi: 10.1038/s42003-025-07557-3 (PMC11807095; doi:10.1038/s42003-025-07557-3)
Supplement: Supplementary file 1 — Supplementary Information [file 42003_2025_7557_MOESM1_ESM.pdf]

SUPPLEMENTARY INFORMATION

Comparison of CRISPR-Cas13b RNA base editing approaches for USH2A-associated inherited retinal degeneration

Authors:

Lewis E. Fry[1,2,3], Lauren Major[1], Ahmed Salman[1], Lucy A. McDermott[1], Jun Yang[4], Andrew J. King[5], Michelle E. McClements[1], and Robert E. MacLaren[1,6]

[1] Nuffield Department of Clinical Neurosciences & NIHR Oxford Biomedical Research Centre, University of Oxford, Oxford, United Kingdom OX3 9DU.

[2] Royal Victorian Eye and Ear Hospital, Victoria, Australia 3002

[3] Centre for Eye Research Australia, Victoria, Australia 3002

[4] Department of Ophthalmology and Visual Sciences, John A. Moran Eye Center, University of Utah, Salt Lake City, UT 84132, USA.

[5] Department of Physiology, Anatomy and Genetics, University of Oxford, United Kingdom.

[6] Oxford Eye Hospital, Oxford University Hospitals NHS Foundation Trust, Oxford, United Kingdom OX3 9DU.

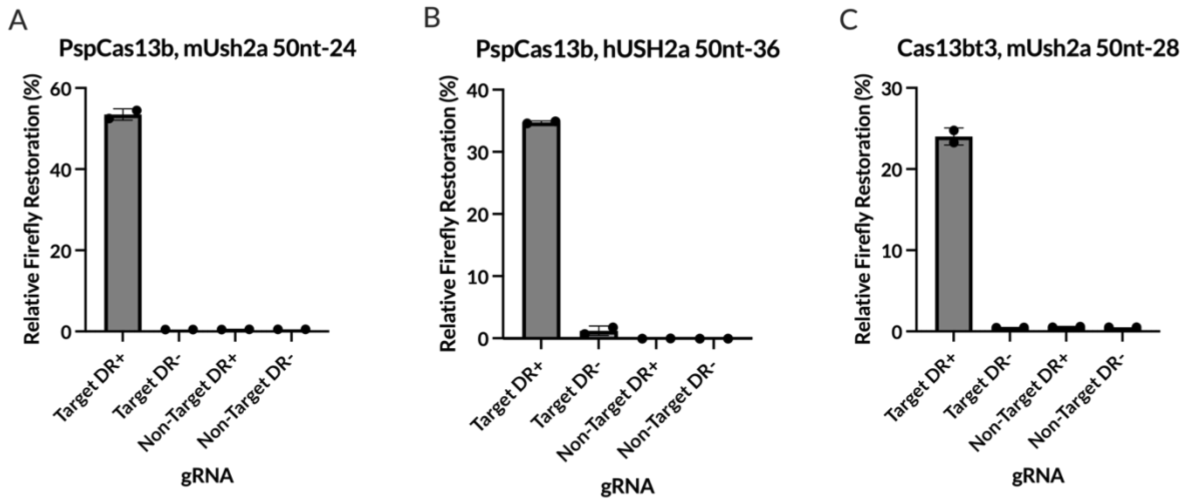

Supplementary Figure 1 | Lack of guide RNA direct repeat abolishes editing
The dual-luciferase assay conducted in HEK293T cells (as in figure 1) was performed to compare gRNAs that had the hairpin-loop direct repeat (DR+) to facilitate interaction with Cas13, and with gRNAs with no direct repeat (DR-). For both PspCas13b targeting either the mUsh2a target or the hUSH2a target (A, B) or with Cas13bt3 targeting mUsh2a (C), editing activity was abolished by removal of the direct repeat from the gRNA. Data shown as mean ± SD, n = 2.

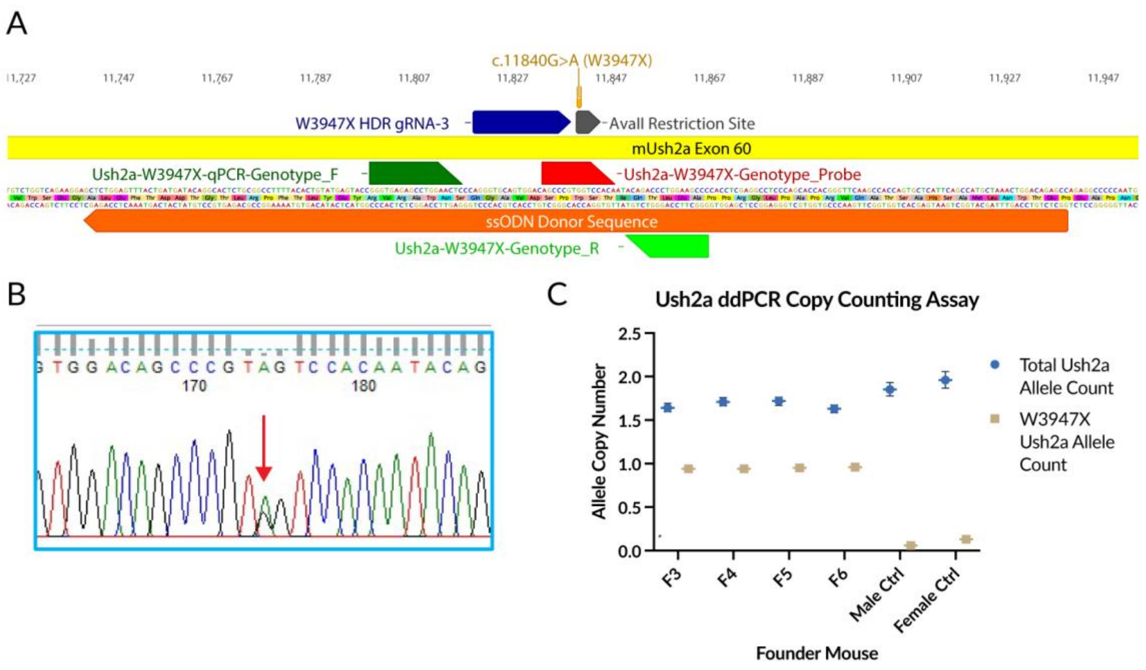

Supplementary Figure 2 | Generation and genotyping of *Ush2a*$^{W3947X/W3947X}$ mouse

A. Locus map of *Ush2a* exon 60. The gRNA sequence (blue) designed to generate a double stranded break for subsequent homology directed repair from the single stranded oligo donor nucleotide (ssODN) template sequence containing the c.118640G>A (p.W3947X) mutation is annotated. This mutation abolishes an AvaII restriction site in the WT sequence. Primers and probes for allelic discrimination genotyping assay are annotated. **B**. Sanger sequencing of founder F_1 heterozgyote performed by MRC Harwell. **C.** ddPCR copy counting assay of founder F1 offspring (labelled F3-F6) showing a single copy of the mutant allele and no more than two copies of *Ush2a*

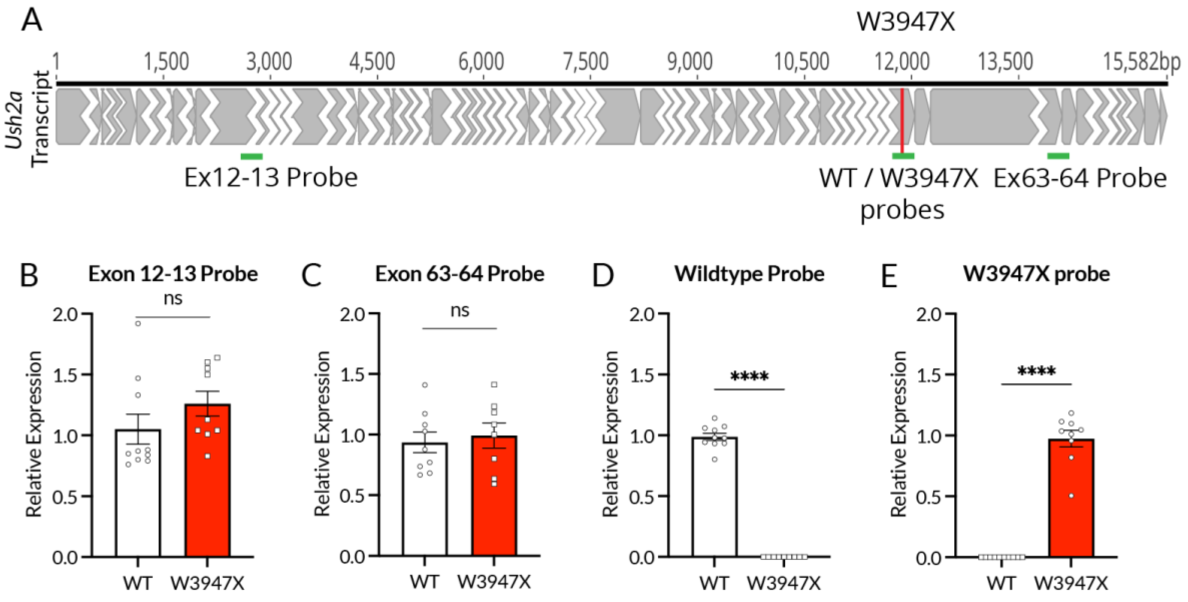

Supplementary Figure 3 | *Ush2a* mRNA expression in retinal lysates *Ush2a*$^{W3947X/W3947X}$ mice

A. Schematic diagram of the *Ush2a* transcript with each grey box representing an exon and a red line showing the W3947X mutation in exon 60. Locations of the exon spanning probes used for qPCR across exon 12-13 and exon 63-64 are shown, along with mutation specific probes to the wildtype or W3947X mutation. **B, C**. Both wildtype and *Ush2a*$^{W3947X/W3947X}$ mice show similar levels of *Ush2a* expression when measured with probes to either exon 12-13 or exon 63-64, indicating mutant transcripts do not undergo non-sense mediated decay. Statistics with unpaired t-test. **D.** No expression of wildtype *Ush2a* is seen in the *Ush2a*$^{W3947X/W3947X}$. **E.** No expression of mutant *Ush2a* is seen in the wildtype mouse. WT n = 10, W3947X n = 9. Unpaired t-tests, **** $p < 0.0001$. Data all mean ± SEM.

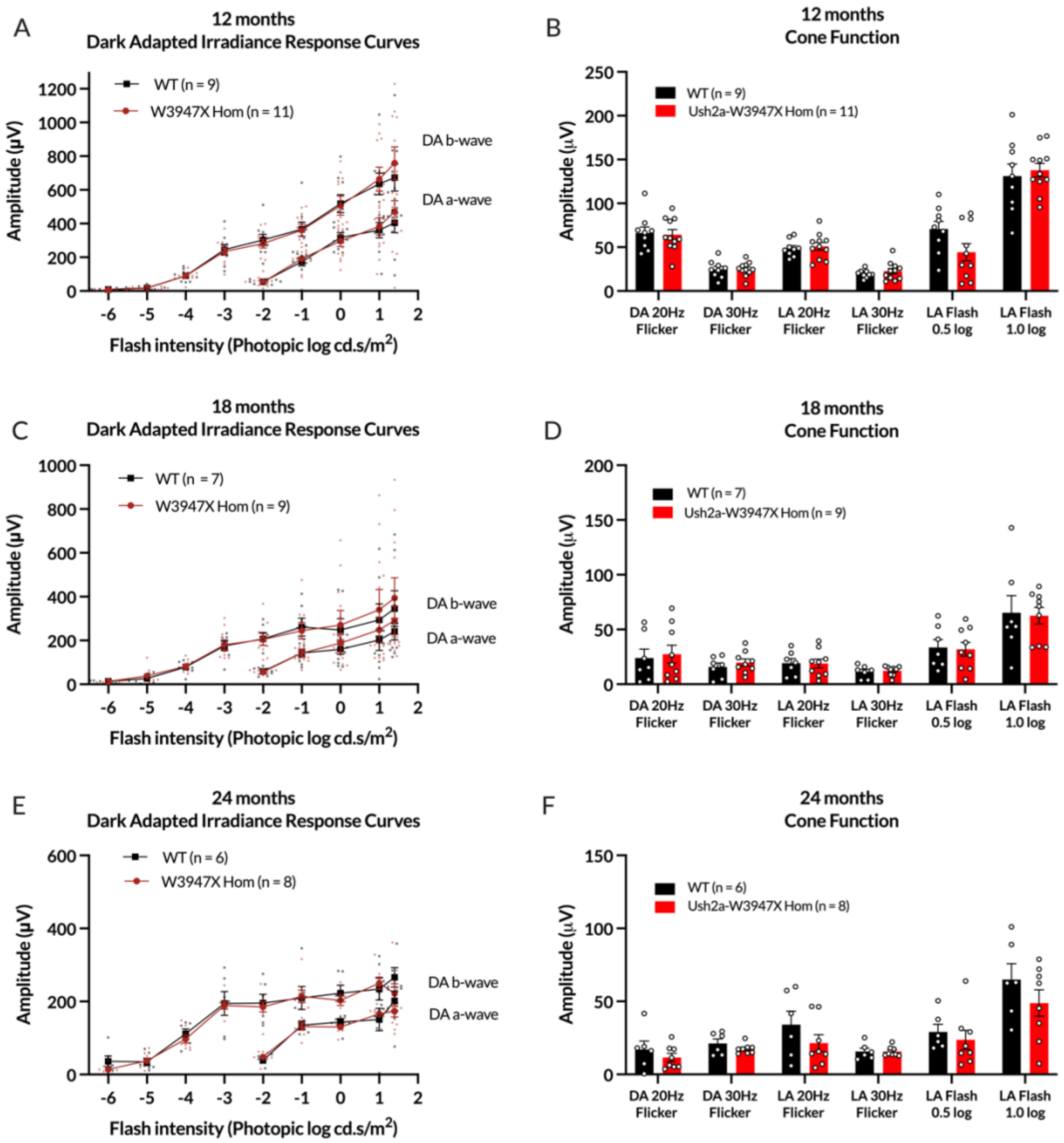

Supplementary Figure 4 | Natural history of retinal function of *Ush2a*$^{W3947X/W3947X}$

No differences in electroretinogram responses are seen between aged WT and *Ush2a*$^{W3947X/W3947X}$ mice at 12-, 18- or 24-months of age. No difference in amplitude in the dark-adapted B-wave or dark-adapted A-wave responses over a range of flash intensities. Measures of cone function including dark-adapted and light-adapted flicker responses, and B-wave amplitudes from photopic flash responses did not show any differences. Statistics with two-way ANOVA with Sidak's multiple comparison testing. Group sizes as indicated in figure legends (n = 6-11). Data all mean ± SEM.

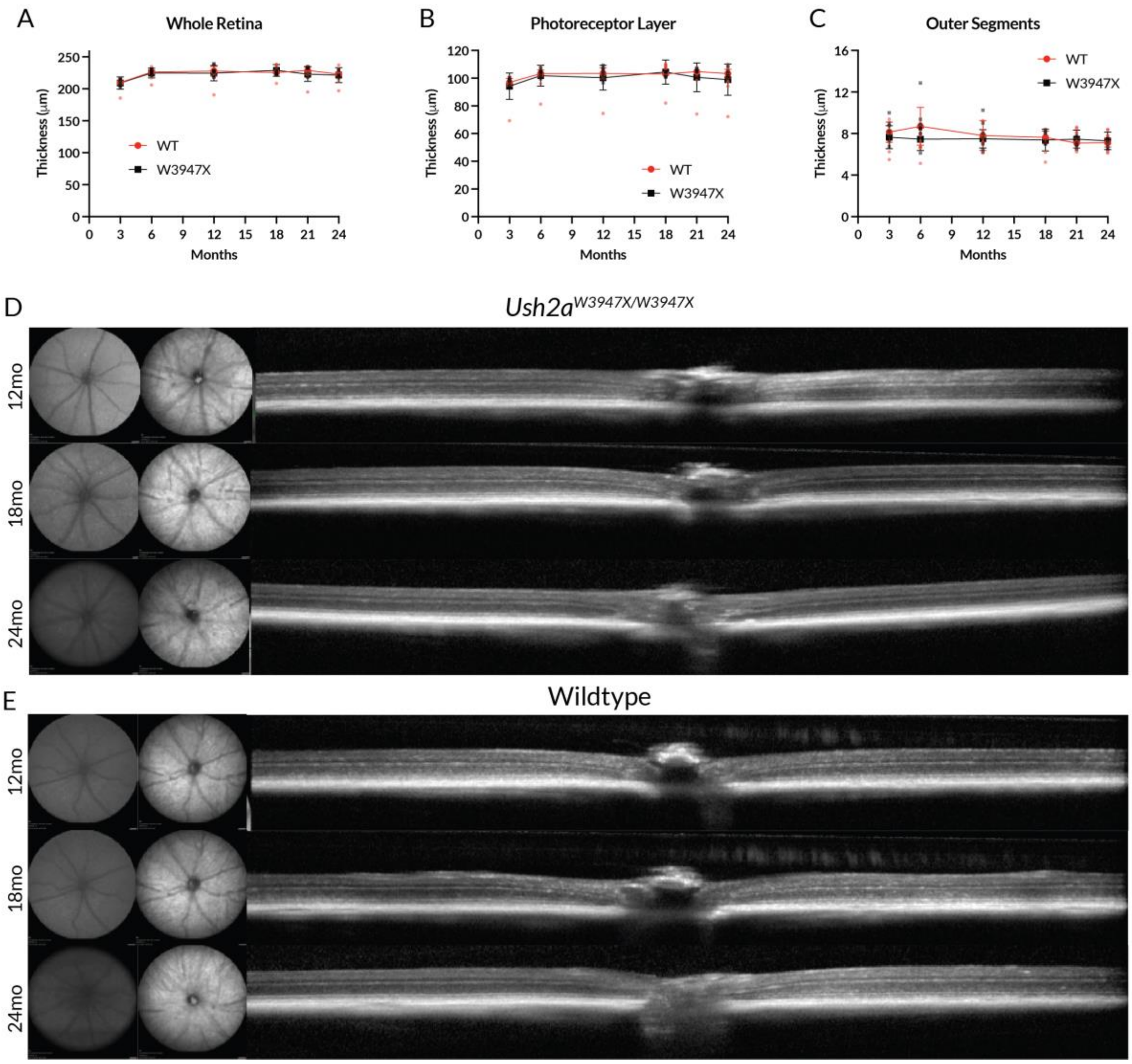

Supplementary Figure 5 | Natural history of retinal structure of *Ush2a*$^{W3947X/W3947X}$

Thickness of the whole retina (A), photoreceptor layer complex (B) and outer segments (C) of *Ush2a*$^{W3947X/W3947X}$ (n = 9) and wildtype mice (n = 7) do not demonstrate differences with age. Representative blue autofluorescence (BAF), infrared reflectance (IR) and OCT images through the optic nerve head from *Ush2a*$^{W3947X/W3947X}$ (D) and wildtype (E) mice. Statistics with two-way ANOVA with Sidak's multiple comparison testing. Data all mean ± SEM.

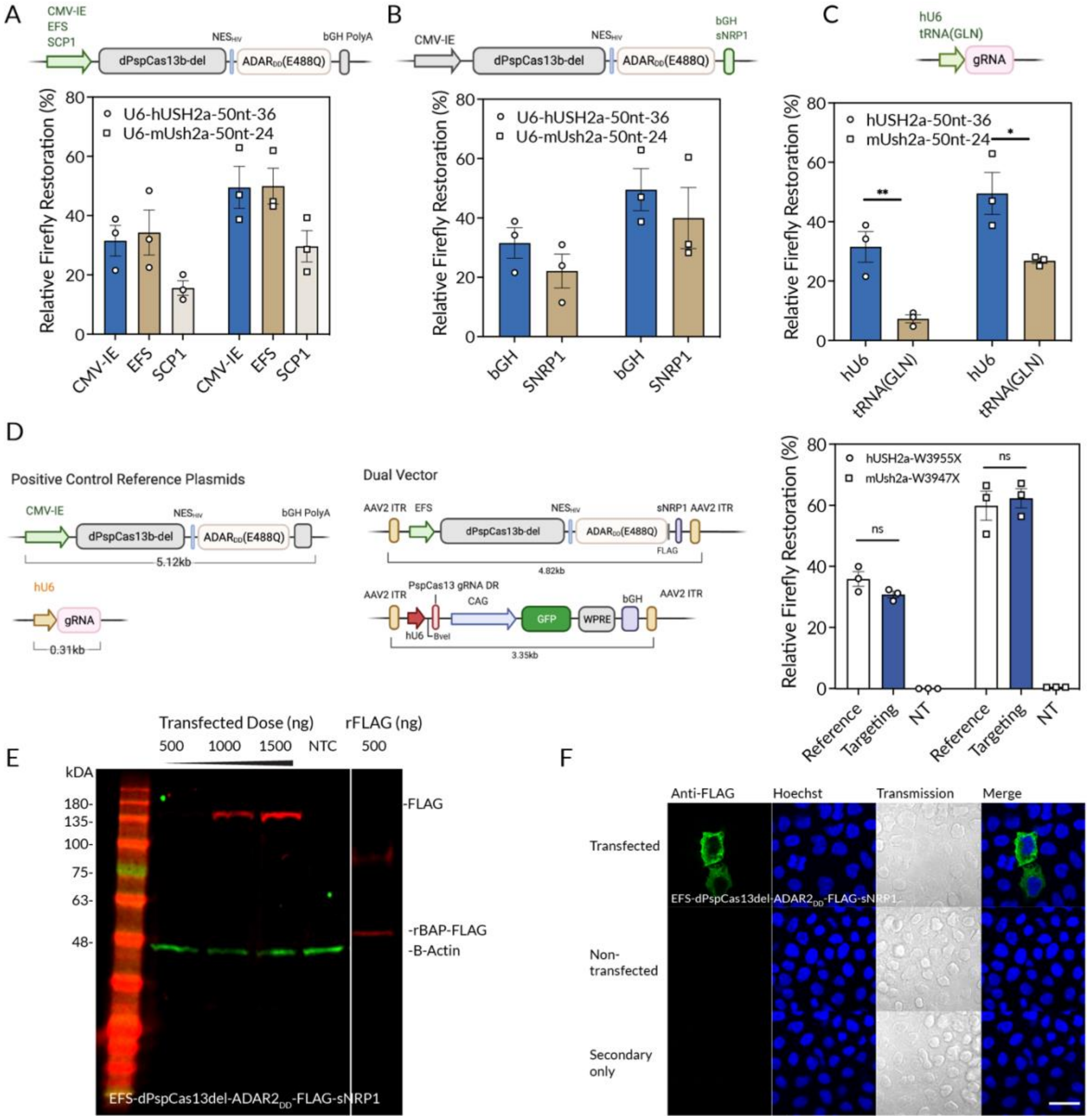
A
CMV-IE
EFS
SCP1
NESHIV
bGH PolyA
dPspCas13b-del
ADARDD(E488Q)
Relative Firefly Restoration (%)
U6-hUSH2a-50nt-36
U6-mUsh2a-50nt-24
CMV-IE
EFS
SCP1
B
CMV-IE
bGH
sNRP1
bGH
SNRP1
C
hU6
tRNA(GLN)
gRNA
hUSH2a-50nt-36
mUsh2a-50nt-24
**
*
D
Positive Control Reference Plasmids
5.12kb
0.31kb
Dual Vector
AAV2 ITR
EFS
FLAG
4.82kb
PspCas13 gRNA DR
CAG
GFP
WPRE
hU6
BveI
3.35kb
hUSH2a-W3955X
mUsh2a-W3947X
ns
Reference
Targeting
NT
E
Transfected Dose (ng)
500
1000
1500
NTC
rFLAG (ng)
kDA
180-
135-
100-
75-
63-
48-
-FLAG
-rBAP-FLAG
-B-Actin
EFS-dPspCas13del-ADAR2DD-FLAG-sNRP1
F
Anti-FLAG
Hoechst
Transmission
Merge
Transfected
Non-transfected
Secondary only
EFS-dPspCas13del-ADAR2DD-FLAG-sNRP1

Supplementary Figure 6 | Testing of minimal elements for AAV-dPspCas13b-ADAR constructs

Comparison of RNA editing rates when using minimal promoters and polyA elements using luciferase assay in transfect HEK293T. **A.** Comparison of RNA Pol II promoters driving dPspCas13-E488Q. Two-way ANOVA with promoter and gRNA as factors: $F(2, 12) = 6.89$, $p = 0.01$ for effect of promoter; $F(2, 12) = 0.058$, $p = 0.94$ for interaction between guide and promoter. No significant difference on Tukey's multiple comparison testing. **B.** Comparison polyA signals for dPspCas13-E488Q demonstrates efficient editing with the snRP1 polyA. Two-way ANOVA with polyA and gRNA as factors: $F(1, 8) = 1.67$, $p = 0.23$ for effect of polyA; $F(1, 8) = 0.0001$, $p = 0.99$ for interaction between guide and polyA. **C.** Comparison of RNA Pol III promoters driving gRNA demonstrates loss of editing efficiency with a tRNA promoter. Two-way ANOVA with promoter and gRNA as factors: $F(1, 8) = 27.64$, $p = 0.008$ for effect of promoter; $F(1, 8) = 0.029$, $p = 0.87$ for interaction between promoter and guide. **p = 0.009 and *p = 0.014, Tukey's multiple comparison test. All data shown as mean ± SEM, n = 3. Data from the CMV-IE.dPspCas13b-del-$ADAR_{DD}$(E488Q) with a hU6 driven gRNA construct (blue bars) used as a reference and displayed in A, B and C. Plasmid maps displayed over graph, with variable region in green. **D.** Comparison of dual vector plasmid constructs with minimal elements for AAV-packaging with original (reference) plasmids used in gRNA screening shown in plasmid maps. Equivalent editing rates were seen between constructs for both guides, without loss of editing efficiency from the weaker EFS promoter and sNRP1 terminator (Two-way ANOVA between targeting guides with construct and gRNA as factors, $F(1,8)=0.18$, $p=0.68$ for effect of construct). **E.** Western blot of protein lysates from cells in 12-well plates transfected with increasing doses of EFS-dPspCas13bdel-$ADAR2_{DD}$-sNRP1. Transgene expression detected with anti-FLAG staining to c-terminal flag tag with the appropriate predicted size (~160kDA, red). A c-terminal FLAG tagged recombinant bacterial alkaline phosphatase (rBAP) protein (~49kDA) was used as a positive control, with β-actin as a protein loading control (green). **F.** Immunocytochemistry following transfection with the AAV-EFS-dPspCas13bdel-$ADAR2_{dd}$-sNRP1 construct. dPspCas13bdel-$ADAR2_{DD}$ expression detected by anti-FLAG staining is observed in the cytoplasmic compartment, as expected due to the fused HIV-derived nuclear export signal. Elements of this figure were created in BioRender. MacLaren (2025) https://BioRender.com/ t62o482, d33o249, b91d116

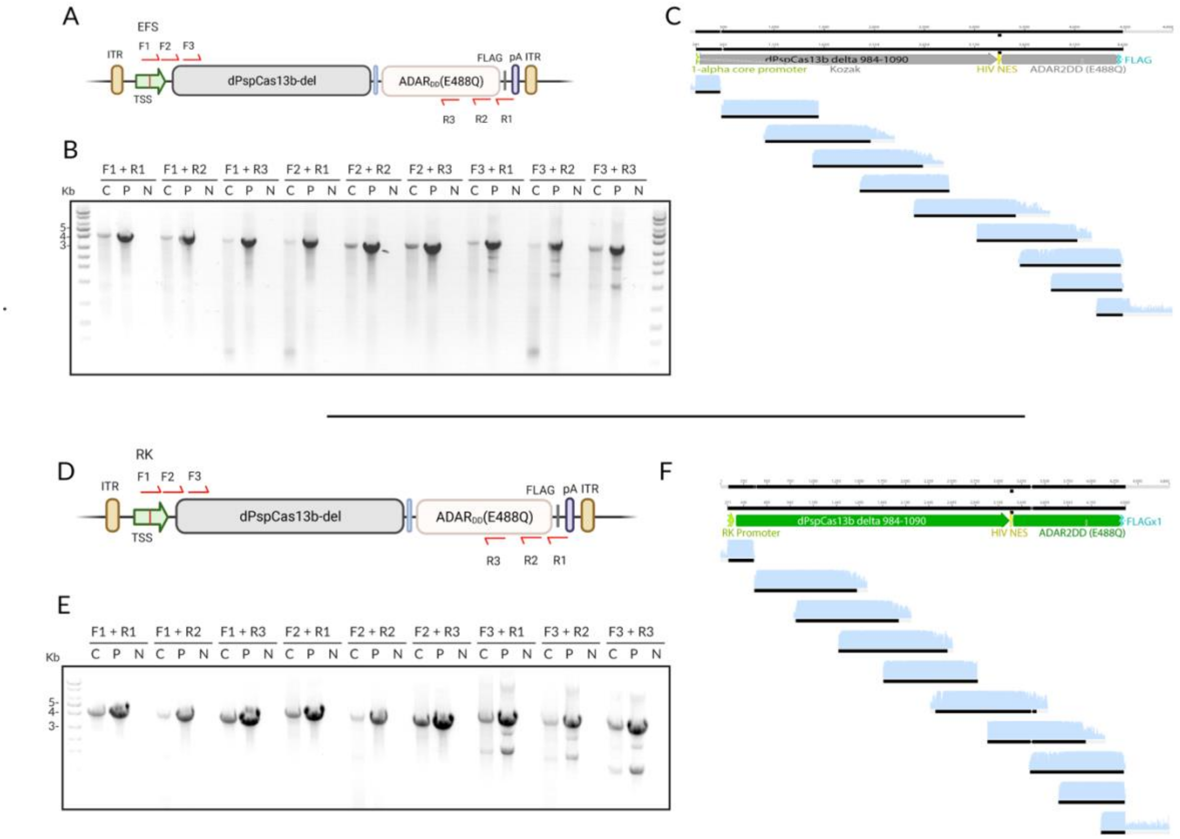

Supplementary Figure 7 | Transcript analysis of dPspCas13-E488Q injected retinas

A. Vector map of AAV-EFS-dPspCas13b-E488Q showing locations of primer pairs. **B.** Analysis using RT-PCR with primer pairs targeting the transcriptional start site (TSS) of the EFS promoter and the beginning of the polyA tail, with further pairs walked in along the transgene. No deletions were observed in products amplified from cDNA (C) compared to plasmid DNA (P). Amplification was not observed in the no-template control (NTC, N). **C.** Sanger sequencing coverage plot of the F1 + R1 RT-PCR product with full coverage and no errors detected. **D.** Vector map of AAV-RK-dPspCas13b-E488Q showing locations of primer pairs. **E.** Similar RT-PCR analysis as in B, of the AAV-RK-dPspCas13b-E488Q vector. **F.** Sanger sequencing coverage plot of the F1 + R1 RT-PCR product with full coverage and no errors detected. n = 2 per construct. Gels are presented uncropped. Elements of this figure were created in BioRender. MacLaren (2025) https://BioRender.com/ h51y722, d40h898

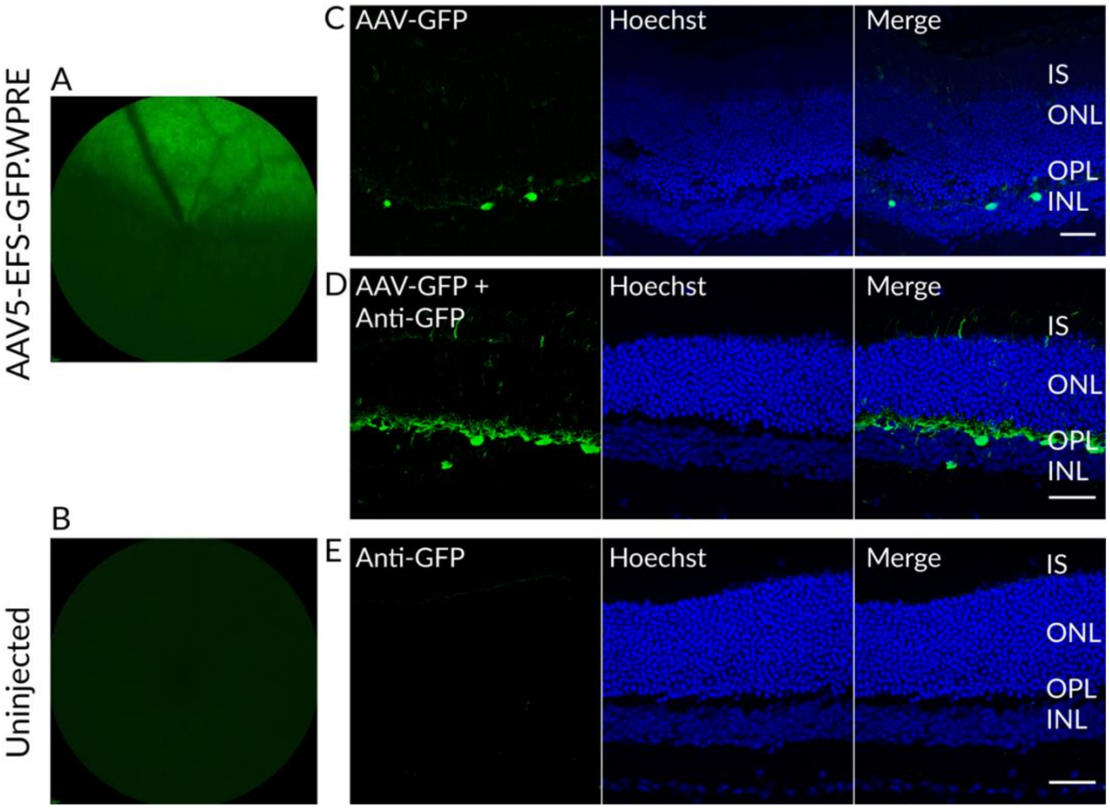

Supplementary Figure 8 | *In vivo* activity of EFS promoter

A-B. *In vivo* CSLO imaging with blue autofluorescence (BAF) demonstrating superior retinal transduction at 3 weeks post-injection with an AAV5-EFS-GFP.WPRE vector (1E+9 gc/eye) (n = 1). **C**. Microscopy of retinal section counterstained with Hoechst. High exposure settings were required to visualise AAV expressed GFP without secondary immunolabelling for signal amplification. **D**. Immunolabelling with anti-GFP antibodies demonstrates GFP expression predominantly in the outer plexiform layer (OPL) with a pattern consistent with expression in horizontal cells. Occasional GFP positive photoreceptor nuclei in the outer nuclear layer (ONL) and inner segments (IS) are seen. **E**. GFP expression is not observed in the un-injected eye. BAF images acquired at 100 detector sensitivity.

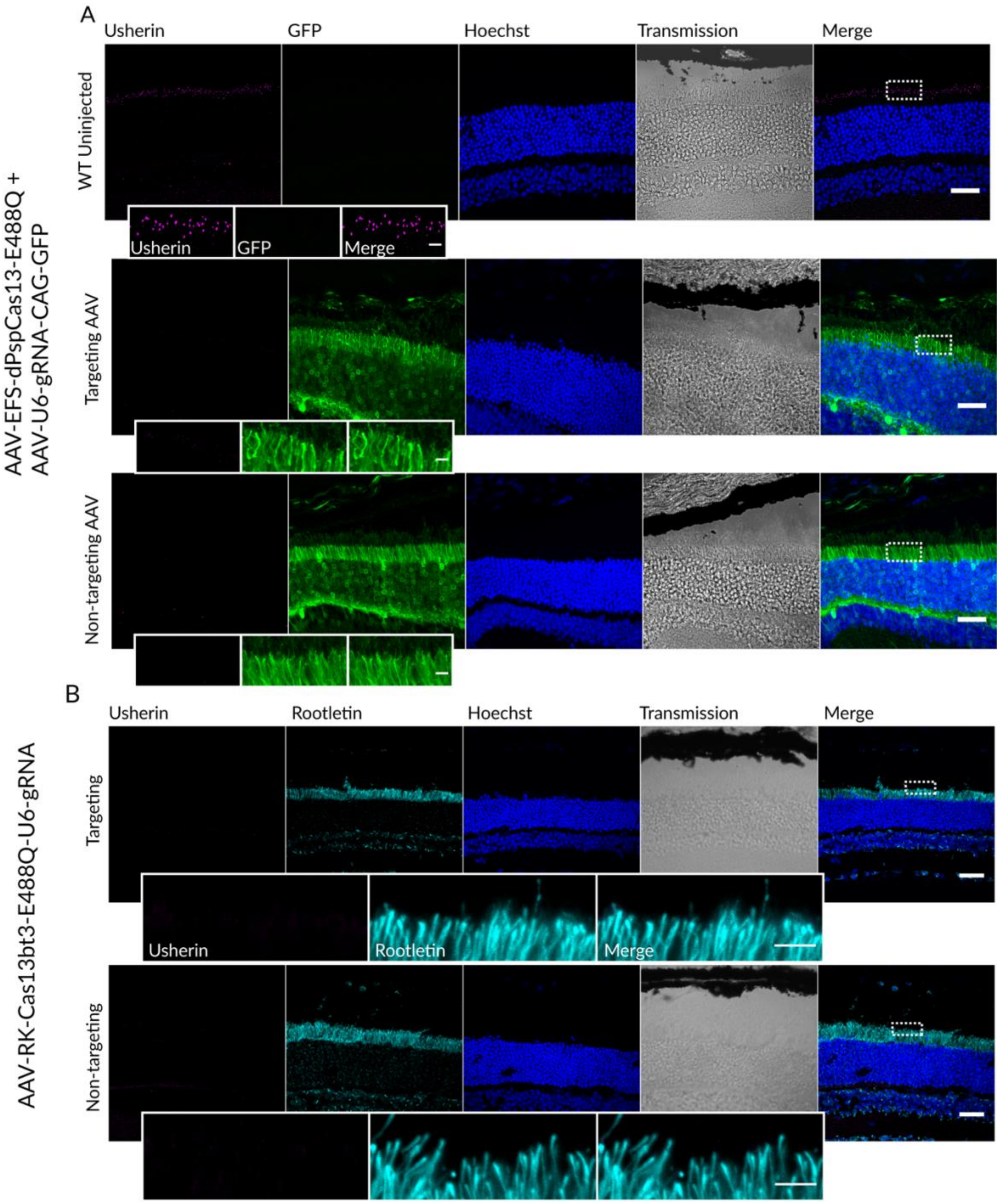

Supplementary Figure 9 | Immunofluorescence images of retinal sections injected with EFS-dPspCas13b-E488Q and RK-dCas13bt3-E488Q AAV vectors

A. Representative retinal immunofluorescence images from mice injected with EFS-dPspCas13b-E488Q vectors, at 40x (scale = 30µm) with inset 63x images focused on the distal inner segment (scale = 5µm). Endogenous GFP fluorescence from the gRNA-GFP vector is seen at the injection site in treated animals. Clear usherin labelling is observed in the wildtype control but not in the AAV injected animals.

B. Representative retinal immunofluorescence images from mice injected with RK-dCas13bt3-E488Q vectors. Usherin restoration is not observed. (n = 2 per group)

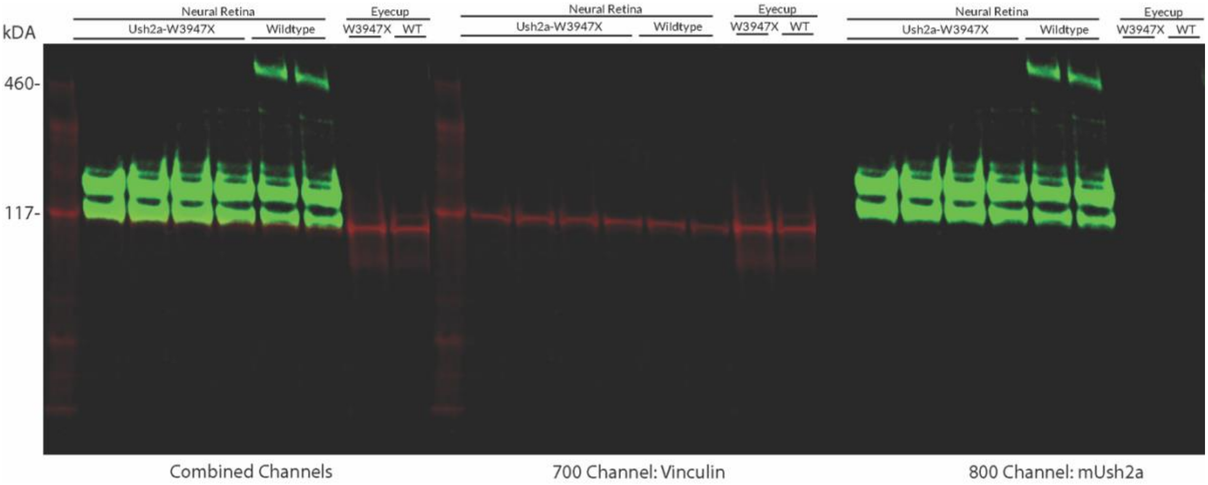

Supplementary Figure 10 | Uncropped blot, Figure 2

Uncropped western blot from Figure 2A showing combined and separate imaging channels. Non-specific shorter bands were observed using the Ush2a antibody in retinal lysates in both the wildtype and mutant mice.

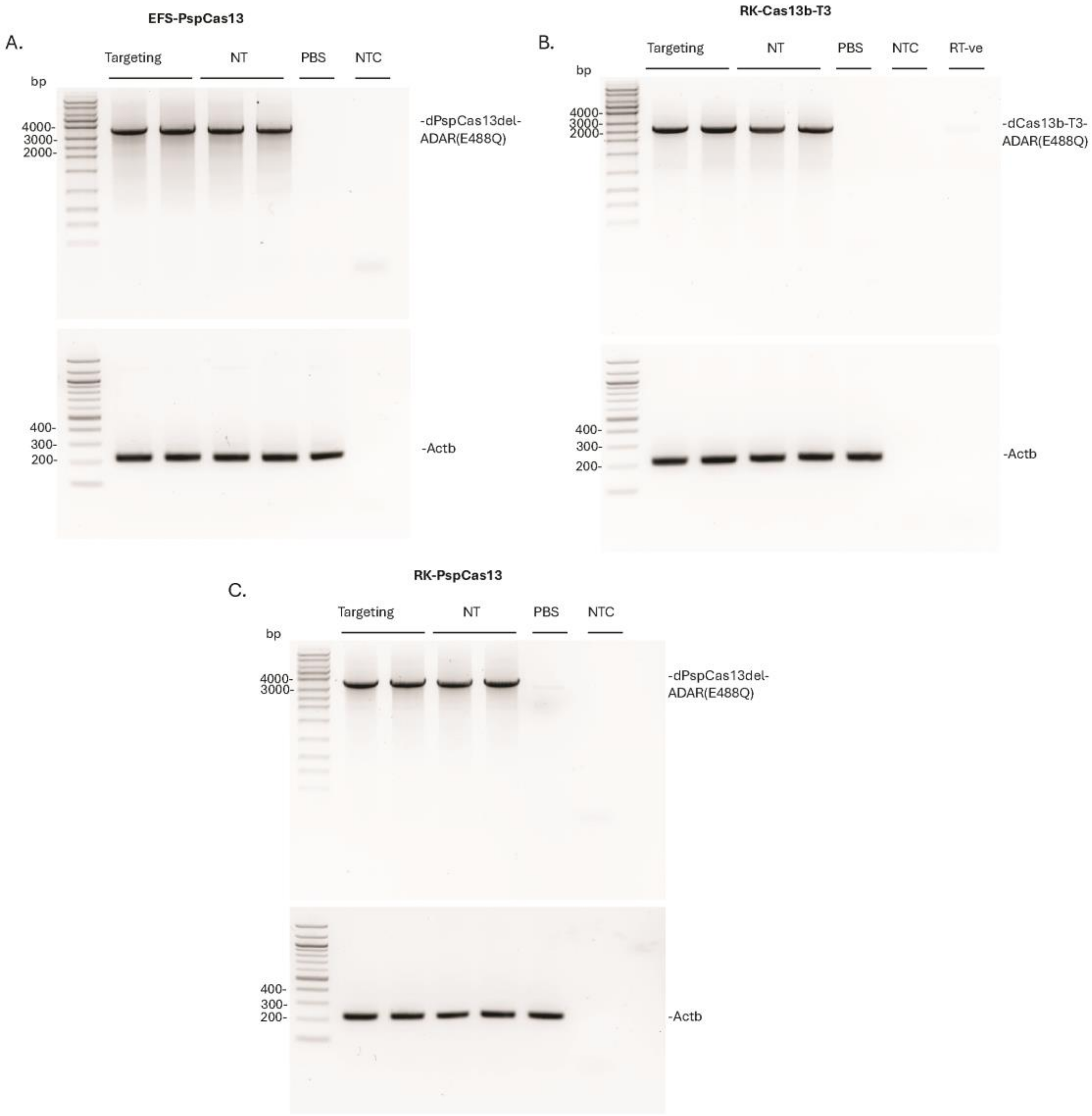

Supplementary Figure 11 | Uncropped gel, Figure 4D

Uncropped reverse transcription PCR gels for each construct, as show in figure 4D.

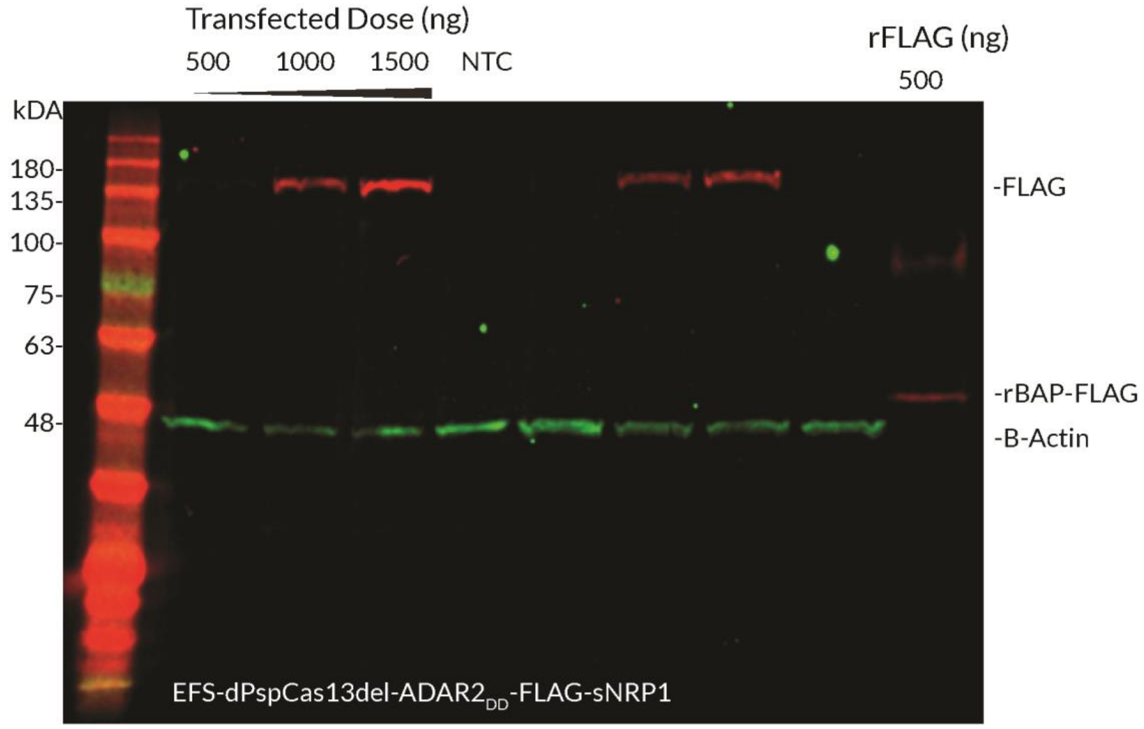

Supplementary Figure 12 | Uncropped blot, Supplementary Figure 6
Uncropped western blot from Supplementary Figure 6E

Full Construct Name	Source
pC0048-CMV-IE-dPspCas13b-longlinker-ADAR2DD(E488Q)	103864
pC0053-CMV-IE-dPspCas13b-GS-ADAR2DD(E488Q)-delta-984-1090	103869
pC0054-CMV-IE-dPspCas13b-longlinker-ADAR2DD(E488Q/T375G)	103870
pC0055-CMV-IE-dPspCas13b-GS-ADAR2DD(E488Q/T375G)-delta-984-1090	103871
pC0043-U6-PspCas13b crRNA backbone	103854
pC0052-REPAIR non-targeting guide clone into pC0043	103868
CMV-IE-HIVNES-dCas13bt1-ADAR2DD(E488Q)-bGH	F. Zhang
CMV-IE-HIVNES-dCas13bt3-ADAR2DD(E488Q)-bGH	F. Zhang
CMV-IE-HIVNES-dCas13bt5-ADAR2DD(E488Q)-bGH	F. Zhang
Bt1-cRNA	F. Zhang
Bt3-cRNA	F. Zhang
Bt5-cRNA	F. Zhang
pSGD Luc 3.0	119760
pX601-AAV-CMV::NLS-SaCas9-NLS-3xHA-bGHpA;U6::BsaI-sgRNA	61591

Supplementary Table 1 | Plasmids used in study
List and origin plasmids used with source or Addgene reference number listed.

Name	Sequence 5'-3'
**Double-stranded DNA fragments**	
hUSH2A-W3955X *Luciferase assay target cassette*	*GATCGAT* ACTCGAGC GCC CTT GAA TTT ATG GAT GAA GGA GAC ACC CTG AGG CCT TTC ACA CTC TAC GAA TAT CGG GTC AGA GCC TGT AAC TCC AAG GGT TCA GTG GAG AGT CTG T**A**G TCA TTA ACA CAA ACT CTG GAA GCT CCA CCT CAA GAT TTT CCA GCT CCT TGG GCT CAA GCC ACG AGT GCT CAT TCA GTT CTG TTG AAT TGG ACA AAG AGATCT *GATCGAT*
mUsh2a-W3947X *Luciferase assay target cassette*	*GATCGAT* ACTCGAGC CTG GAG TTT ACT GAT GAT ACA GGC ACT CTG CGG CCT TTT ACA CTG TAT GAG TAC CGG GTG AGA GCC TGG AAC TCC CAG GGT GCA GTG GAC AGC CCG T**A**G TCC ACA ATA CAG ACC CTG GAA GCC CCA CCT CG***T*** GGC CTC CCA GCA CCA CGG GTT CAA GCC ACC AGT GCT CAT TCA GCC ATG CTA AAC TGG ACA GAG CCA AGATCT *GATCGAT*
Italicised sequences are stuffer nucleotides to enable restriction enzyme binding or overlap sequences for multifragment assembly, underlined nucleotides are restriction enzyme sites. Grey highlight indicates the promoter or polyA sequence. The target mutation is highlight in bold red. A silent A>T mutation was introduced into the mUsh2a-W3947X sequence to abolish a PspXi site for cloning (italicised, underlined). Bold sequences indicated gRNA direct repeats.	
**gRNA Sequences**	
PspCas13b guides	
5' overhangs, U6 promoter	FW: CACC<br>RV: CAAC
5' overhangs, tRNA(GLN) promoter	FW: ACCT<br>RV: CAAC
mUsh2a-G1-32<br>5'G added	GAGGGTCTGTATTGTGGAC**C**ACGGGCTGTCCACTGCACCCTGGGAGTTCCA
mUsh2a-G2-34	GGTCTGTATTGTGGAC**C**ACGGGCTGTCCACTGCACCCTGGGAGTTCCAGG
mUsh2a-G3-36<br>5'G added	GTCTGTATTGTGGAC**C**ACGGGCTGTCCACTGCACCCTGGGAGTTCCAGGCT
mUsh2a-G4-26	GCTTCCAGGGTCTGTATTGTGGAC**C**ACGGGCTGTCCACTGCACCCTGGGA
mUsh2a-G5-42<br>5'G added	GTTGTGGAC**C**ACGGGCTGTCCACTGCACCCTGGGAGTTCCAGGCTCTCACC
mUsh2a-G6-16<br>5'G added	GACGAGGTGGGGCTTCCAGGGTCTGTATTGTGGAC**C**ACGGGCTGTCCACTG
mUsh2a-G7-18<br>5'G added	GGAGGTGGGGCTTCCAGGGTCTGTATTGTGGAC**C**ACGGGCTGTCCACTGCA
mUsh2a-G8-20	GGTGGGGCTTCCAGGGTCTGTATTGTGGAC**C**ACGGGCTGTCCACTGCACC
mUsh2a-G9-22<br>5'G added	GTGGGGCTTCCAGGGTCTGTATTGTGGAC**C**ACGGGCTGTCCACTGCACCCT

mUsh2a-G10-24	GGGCTTCCAGGGTCTGTATTGTGGAC**C**ACGGGCTGTCCACTGCACCCTGG
mUsh2a-G11-28<br>5'G added	GTTCCAGGGTCTGTATTGTGGAC**C**ACGGGCTGTCCACTGCACCCTGGGAGT
mUsh2a-G12-30<br>5'G added	GCCAGGGTCTGTATTGTGGAC**C**ACGGGCTGTCCACTGCACCCTGGGAGTTC
mUsh2a-G13-38<br>5'G added	GTGTATTGTGGAC**C**ACGGGCTGTCCACTGCACCCTGGGAGTTCCAGGCTCT
mUsh2a-G14-40<br>5'G added	GTATTGTGGAC**C**ACGGGCTGTCCACTGCACCCTGGGAGTTCCAGGCTCTCA
mUsh2a-G15-30nt-22<br>5'G added	GTTGTGGAC**C**ACGGGCTGTCCACTGCACCCT
mUsh2a-G16-30nt-24<br>5'G added	GGTGGAC**C**ACGGGCTGTCCACTGCACCCTGG
mUsh2a-G17-30nt-26	GGAC**C**ACGGGCTGTCCACTGCACCCTGGGA
mUsh2a-G18-30nt-28<br>5'G added	GAC**C**ACGGGCTGTCCACTGCACCCTGGGAGT
hUSH2A-G1-32<br>5'G added	GAGAGTTTGTGTTAATGAC**C**ACAGACTCTCCACTGAACCCTTGGAGTTACA
hUSH2A-G2-34<br>5'G added	GAGTTTGTGTTAATGAC**C**ACAGACTCTCCACTGAACCCTTGGAGTTACAGG
hUSH2A-G3-36<br>5'G added	GTTTGTGTTAATGAC**C**ACAGACTCTCCACTGAACCCTTGGAGTTACAGGCT
hUSH2A-G4-18	GAGGTGGAGCTTCCAGAGTTTGTGTTAATGAC**C**ACAGACTCTCCACTGAA
hUSH2A-G5-20	GGTGGAGCTTCCAGAGTTTGTGTTAATGAC**C**ACAGACTCTCCACTGAACC
hUSH2A-G6-22	GTGGAGCTTCCAGAGTTTGTGTTAATGAC**C**ACAGACTCTCCACTGAACCCT
hUSH2A-G7-24	GAGCTTCCAGAGTTTGTGTTAATGAC**C**ACAGACTCTCCACTGAACCCTTG
hUSH2A-G8-26	GCTTCCAGAGTTTGTGTTAATGAC**C**ACAGACTCTCCACTGAACCCTTGGA
hUSH2A-G9-28	GTTCCAGAGTTTGTGTTAATGAC**C**ACAGACTCTCCACTGAACCCTTGGAGT
hUSH2A-G10-30	GCCAGAGTTTGTGTTAATGAC**C**ACAGACTCTCCACTGAACCCTTGGAGTTA
hUSH2A-G11-38	GTGTGTTAATGAC**C**ACAGACTCTCCACTGAACCCTTGGAGTTACAGGCTCT
hUSH2A-G12-40	GTGTTAATGAC**C**ACAGACTCTCCACTGAACCCTTGGAGTTACAGGCTCTGA
hUSH2A-G13-42	GTTAATGAC**C**ACAGACTCTCCACTGAACCCTTGGAGTTACAGGCTCTGACC
Non-targeting-50nt	GTAATGCCTGGCTTGTCGACGCATAGTCTGGTAATGCCTGGCTTGTCGAC

Non-targeting-30nt	GTAATGCCTGGCTTGTCGACGCATAGTCTG
Cas13bt Guides	
5' overhangs, U6 promoter	FW: CACC<br>RV: CAGC
bt-mUsh2a-30nt-14	GGTCTGTATTGTGGAC**C**ACGGGCTGTCCAC
bt-mUsh2a-30nt-16	GTCTGTATTGTGGAC**C**ACGGGCTGTCCACTG
bt-mUsh2a-30nt-18	GTGTATTGTGGAC**C**ACGGGCTGTCCACTGCA
bt-mUsh2a-30nt-20	GTATTGTGGAC**C**ACGGGCTGTCCACTGCACC
bt-mUsh2a-30nt-22	GTTGTGGAC**C**ACGGGCTGTCCACTGCACCCT
bt-mUsh2a-30nt-24	GTGGAC**C**ACGGGCTGTCCACTGCACCCTGG
bt-mUsh2a-30nt-26	GGAC**C**ACGGGCTGTCCACTGCACCCTGGGA
bt-mUsh2a-30nt-28	GAC**C**ACGGGCTGTCCACTGCACCCTGGGAGT
Bt-Non-target-30nt	GTAATGCCTGGCTTGTCGACGCATAGTCTG
bt-mUsh2a-50nt-18<br>5'G added	GGAGGTGGGGCTTCCAGGGTCTGTATTGTGGAC**C**ACGGGC<br>TGTCCACTGCA
bt-mUsh2a-50nt-20	GGTGGGGCTTCCAGGGTCTGTATTGTGGAC**C**ACGGGCTGT<br>CCACTGCACC
bt-mUsh2a-50nt-22<br>5'G added	GTGGGGCTTCCAGGGTCTGTATTGTGGAC**C**ACGGGCTGTC<br>CACTGCACCCT
bt-mUsh2a-50nt-24	GGGCTTCCAGGGTCTGTATTGTGGAC**C**ACGGGCTGTCCAC<br>TGCACCCTGG
bt-mUsh2a-50nt-26	GCTTCCAGGGTCTGTATTGTGGAC**C**ACGGGCTGTCCACTGC<br>ACCCTGGGA
bt-mUsh2a-50nt-28<br>5'G added	GTTCCAGGGTCTGTATTGTGGAC**C**ACGGGCTGTCCACTGCA<br>CCCTGGGAGT
bt-mUsh2a-50nt-30<br>5'G added	GCCAGGGTCTGTATTGTGGAC**C**ACGGGCTGTCCACTGCACC<br>CTGGGAGTTC
bt-mUsh2a-50nt-32	GAGGGTCTGTATTGTGGAC**C**ACGGGCTGTCCACTGCACCCT<br>GGGAGTTCCA
bt-mUsh2a-50nt-34	GGTCTGTATTGTGGAC**C**ACGGGCTGTCCACTGCACCCTGG<br>GAGTTCCAGG
bt-mUsh2a-50nt-36	GTCTGTATTGTGGAC**C**ACGGGCTGTCCACTGCACCCTGGGA<br>GTTCCAGGCT
bt-mUsh2a-50nt-38<br>5'G added	GTGTATTGTGGAC**C**ACGGGCTGTCCACTGCACCCTGGGAG<br>TTCCAGGCTCT
bt-mUsh2a-50nt-40	GTTGTGGAC**C**ACGGGCTGTCCACTGCACCCTGGGAGTTCCA<br>GGCTCTCACC
Bt-Non-target-50nt	GTAATGCCTGGCTTGTCGACGCATAGTCTGGTAATGCCTGG<br>CTTGTCGAC

All guides were synthesised as single stranded oligonucleotides and annealed to a synthesised complementary sequence for cloning. Appropriate restriction overhangs were added depending on the golden gate cloning destination specified above. A-C mismatch specifying the target nucleotide for editing is bold and underlined, with the mismatch distance specified in the gRNA sequence name. 5' G added to guide for U6 expression if not present.

RT-PCR and sequencing primers for editing detection

Base decomposition, luciferase plasmid

pSGDLuc F2	TTGACCTTCTTAAGCTGGCG
pSGDLuc R1	CTTCGAGTGGGTAGAATGGC

Sequences of Key Plasmid Elements

tRNA(GLN) Promoter	GGTTCCATGGTGTAATGGTTAGCACTCTGGACTCTGAATCC AGCGATCCGAGTTCAAATCTCGGTGGAACCT
SCP1 Promoter	GTA CTT ATA TAA GGG GGT GGG GGC GCG TTC GTC CTC AGT CGC GAT CGA ACA CTC GAG CCG AGC AGA CGT GCC TAC GGA CC
EFS Promoter	GGG CAG AGC GCA CAT CGC CCA CAG TCC CCG AGA AGT TGG GGG GAG GGG TCG GCA ATT GAT CCG GTG CCT AGA GAA GGT GGC GCG GGG TAA ACT GGG AAA GTG ATG TCG TGT ACT GGC TCC GCC TTT TTC CCG AGG GTG GGG GAG AAC CGT ATA TAA GTG CAG TAG TCG CCG TGA ACG TTC TTT TTC GCA ACG GGT TTG CCG CCA GAA CACA
MinCMV.Inr Promoter	GGTAGGCGTGTACGGTGGGAGGCCTATATAAGCAGAGCTC GTTTAGTGAACCGTCAGATC
minCMV.SCP3 Promoter	GGTAGGCGTGTACGGTGGGAGGCCTATATAAGCAGAGCTC GTTTAGTGAACCGTCAGTCCGCCTGGATACATCGAGCCGAG TGGTCGTGCCTCCATAGAA
CMV-IE Promoter	GACATTGATTATTGACTAGTTATTAATAGTAATCAATTACGG GGTCATTAGTTCATAGCCCATATATGGAGTTCCGCGTTACA TAACTTACGGTAAATGGCCCGCCTGGCTGACCGCCCAACGA CCCCCGCCCATTGACGTCAATAATGACGTATGTTCCCATAGT AACGCCAATAGGGACTTTCCATTGACGTCAATGGGTGGAGT ATTTACGGTAAACTGCCCACTTGGCAGTACATCAAGTGTAT CATATGCCAAGTACGCCCCCTATTGACGTCAATGACGGTAA ATGGCCCGCCTGGCATTATGCCCAGTACATGACCTTATGGG ACTTTCCTACTTGGCAGTACATCTACGTATTAGTCATCGCTA TTACCATGGTGATGCGGTTTTGGCAGTACATCAATGGGCGT GGATAGCGGTTTGACTCACGGGGATTTCCAAGTCTCCACCC CATTGACGTCAATGGGAGTTTGTTTTGGCACCAAAATCAAC GGGACTTTCCAAAATGTCGTAACAACTCCGCCCCATTGACG CAAATGGGCGGTAGGCGTGTACGGTGGGAGGTCTATATAA GCAGAGCTCTCTGGCTAACTACCGGTGCCACCATGG
CMV Promoter (no enhancer)	GTGATGCGGTTTTGGCAGTACATCAATGGGCGTGGATAGC GGTTTGACTCACGGGGATTTCCAAGTCTCCACCCCATTGAC GTCAATGGGAGTTTGTTTTGGCACCAAAATCAACGGGACTT TCCAAAATGTCGTAACAACTCCGCCCCATTGACGCAAATGG GCGGTAGGCGTGTACGGTGGGAGGTCTATATAAGCAGAGC TCTCTGGCTAACTA

RK -112/+87 promoter	GGGCCCCAGAAGCCTGGTGGTTGTTTGTCCTTCTCAGGGG AAAAGTGAGGCGGCCCCTTGGAGGAAGGGGCCGGGCAGA ATGATCTAATCGGATTCCAAGCAGCTCAGGGGATTGTCTTT TTCTAGCACCTTCTTGCCACTCCTAAGCGTCCTCCGTGACCC CGGCTGGGATTTAGCCTGGTGCTGTGTCAGCCCCGGG
hU6 Promoter	GAGGGCCTATTTCCCATGATTCCTTCATATTTGCATATACGA TACAAGGCTGTTAGAGAGATAATTGGAATTAATTTGACTGT AAACACAAAGATATTAGTACAAAATACGTGACGTAGAAAGT AATAATTTCTTGGGTAGTTTGCAGTTTTAAAATTATGTTTTA AAATGGACTATCATATGCTTACCGTAACTTGAAAGTATTTCG ATTTCTTGGCTTTATATATCTTGTGGAAAGGAC
bGH PolyA	CTGTGCCTTCTAGTTGCCAGCCATCTGTTGTTTGCCCCTCCC CCGTGCCTTCCTTGACCCTGGAAGGTGCCACTCCCACTGTCC TTTCCTAATAAAATGAGGAAATTGCATCGCATTGTCTGAGT AGGTGTCATTCTATTCTGGGGGGTGGGGTGGGGCAGGAC AGCAAGGGGGAGGATTGGGAAGACAATAGCAGGCATGCT GGGGATGCGGTGGGCTCTATGG
SV40 PolyA	TAAGATACATTGATGAGTTTGGACAAACCACAACTAGAATG CAGTGAAAAAAATGCTTTATTTGTGAAATTTGTGATGCTAT TGCTTTATTTGTAACCATTATAAGCTGCAATAAACAAGTT
sNRP1 PolyA (Dual)	AAAATAAAATACGAAATGAAATAAAATACGAAATG
**Mouse Generation**	
gRNA W3947X-HDR-g3	AGGGTGCAGTGGACAGCCCG
ssDNA: W3947X HDR	GGCTCTGTCCAGTTTAGCATGGCTGAATGAGCACTGGTGG CTTGAACCCGTGGTGCTGGGAGGCCTCGAGGTGGGGCTTC CAGGGTCTGTATTGTGGACCACGGGCTGTCCACTGCACCCT GGGAGTTCCAGGCTCTCACCCGGTACTCATACAGTGTAAAA GGCCGCAGAGTGCCTGTATCATCAGTAAACTCCAGAGC
**Mouse Genotyping**	
Allelic Discrimination Assay	
Ush2a-W3947X_F *Common FW Primer*	GGGTGAGAGCCTGGAACTC
Ush2a-W3947X _WT_PROBE	5'-FAM-AGCCCGTGGTCCACA-BHQplus-3'
Ush2a-W3947X _Mutant_PROBE	5'-TET-AGCCCGTAGTCCACA-BHQplus-3'
Ush2a-W3947X _R *Common RV Primer*	GGGCTTCCAGGGTCTGT
ddPCR Assay	
Universal Ush2a Assay	
FW Primer	GCTCTGGAGTTTACTGATGATACA
RV Primer	CAATACAGACCCTGGAAGCC
Probe	5'-FAM-TGTATGAGTACCGGGTGAGAGCCT

Ush2a-W3947X Assay	
FW Primer	GCAGTGGACAGCCCGTA
RV Primer	TGCTCATTCAGCCATGCTAAAC
Probe	5'-FAM-CCACAATACAGACCCTGGAAGCCC
Dot1l Reference Assay	
FW Primer	GCCCCAGCACGACCATT
RV Primer	TAGTTGGCATCCTTATGCTTCATC
Probe	5'-FAM-CCCAACAGGCCTGGATTCTCAATGC
**AAV Titre Primers**	
PspCas13 AAVs	
AAVtitre-Cas13-F4	AGAACAGAGCGGTACAGAAAG
AAVtitre-Cas13-R4	CCAGGATTGTCTCGATCTCTTC
Cas13bt3 AAVs and gRNA-GFP AAVs	
AAVtitre-U6-F5	CGATACAAGGCTGTTAGAGAGATA
AAVtitre-U6-R5	AAACTGCAAACTACCCAAGAAA
**On-target editing amplification and sequencing**	
Sanger Sequencing: Amplification and sequencing	
mUsh2a-ex60-F1	TATGTCGTTCACAGGCGTCC
mUsh2a-ex60-R1	TTCCCGTCACCGTGAAAGC
PCR primers, targeted deep sequencing – partial illumine adapters underlined	
mUsh2a-ex60-F1-NGS	ACACTCTTTCCCTACACGACGCTCTTCCGATCTTATGTCGTTCACAGGCGTCC
mUsh2a-ex60-R1-NGS	GACTGGAGTTCAGACGTGTGCTCTTCCGATCTTTCCCGTCACCGTGAAAGC
**Transgene Amplification**	
EFS-PspCas13b Forward Primers	
F1-EFS	TTTGCCGCCAGAACACAG
RK-PspCas13b Forward Primers	
F1-RK	CCTTCTTGCCACTCCTAAGC

RK-Cas13bt3 Forward Primers	
F1-bt3*	GCGACAGAATTACAACAG
Universal PspCas13b Forward Primers	
F2*	CTTAAGCTTGCCACCATGAAC
F3	GATGAGCCACCTGTACAACG
Universal ADAR2$_{DD}$ Reverse primers	
R1	ATCAGCGAGCTCTAGGAATTC
R2	GTACTCCTTTGCCGCCAG
R3	TGCTCGAGAAGTAAATGGGC

Supplementary Table 2 | Oligonucleotide sequences used in the study

Step	Description	Photopic Log cd.s/m²	Background (cd/m²)	Sweeps (n)	Intersweep Delay (ms)	Pulse Frequency (Hz)	Pre-step Adaption Time (s)
**1**	Single 0.5Hz $1x10^{-6}$ cd.s/m²	1e-6	0	16	2000	0.5	0
**2**	Single 0.5Hz $1x10^{-5}$ cd.s/m²	1e-5	0	16	2000	0.5	5
**3**	Single 0.5Hz $1x10^{-4}$ cd.s/m²	1e-4	0	9	5000	0.5	10
**4**	Single 0.5Hz $1x10^{-3}$ cd.s/m²	1e-3	0	9	5000	0.5	30
**5**	Single 0.5Hz $1x10^{-2}$ cd.s/m²	1e-2	0	9	5000	0.5	30
**6**	Single 0.5Hz $1x10^{-1}$ cd.s/m²	1e-1	0	4	20000	0.5	30
**7**	Single 0.5Hz 1.0 cd.s/m²	0	0	4	20000	0.5	45
**8**	Single 0.5Hz $1x10^{1}$ cd.s/m²	1	0	2	40000	0.5	90
**9**	Single 0.5Hz 25 cd.s/m²	1.4	0	1	0	0.5	180
**10**	Continuous 10Hz 3.0 cd.s/m²	0.5	0	20	0	10	60
**11**	Continuous 20Hz 3.0 cd.s/m²	0.5	0	20	0	20	10
**12**	Continuous 30Hz 3.0 cd.s/m²	0.5	0	20	0	30	10
	Light adaption						600
**13**	Single 0.5Hz 1.0 cd.s/m²	0.5	30	10	1000	0.5	5
**14**	Continuous 20hz 3.0 cd.s/m²	0.5	30	20	0	20	5
**15**	Single 0.5Hz 10 cd.s/m²	1	30	5	1000	0.5	5

Supplementary Table 3 | Electroretinography recording protocol
Standard custom ERG recording protocol programmed with Espion software (Diagnosys, UK)

Antibody	Dilution	Host	Application	Source
**Primaries**				
USH2A (C-terminal)	1:4000*	Rabbit	WB<br>IHC	Gift from Dr. Jun Yang, University of Utah
Vinculin	1:200	Mouse	WB	Sigma-Aldrich (V9131)
FLAG M2	1:1000	Mouse	WB, IHC	Sigma-Aldrich (F1804)
Rootletin	1:400	Chicken	IHC	MerckMillipore (ABN1686)
Phalloidin (Alexa Fluor 488)	1:40	Amanita phalloides	IHC	Thermo Fisher Scientific (A12379)
GFP	1:500	Rabbit	IHC	Sigma-Aldrich (G1544)
**Secondaries**				
Donkey anti-rabbit 800W	1:10,000		WB	LI-COR Biosciences 926-32213
Donkey anti-mouse 680RD	1:10,000		WB	LI-COR Biosciences 926-68072
AlexaFluor goat anti-chicken 647	1:500		IHC	Thermo Fisher Scientific A32933
AlexaFluor donkey anti-mouse 488	1:500		IHC	Thermo Fisher Scientific A32766
AlexaFluor goat anti-rabbit 488	1:500		IHC	Thermo Fisher Scientific A32731
AlexaFluor goat anti-rabbit 568	1:500		IHC	Thermo Fisher Scientific A-11011

Supplementary Table 4 | Antibodies used for western blot and immunohistochemistry
*Used at 1:2000 for cochlea wholemounts
