# Supplementary material for: Comparison of CRISPR-Cas13b RNA base editing approaches for USH2A-associated inherited retinal degeneration
Source: Commun Biol. 2025 Feb 8;8:200. doi: 10.1038/s42003-025-07557-3 (PMC11807095; doi:10.1038/s42003-025-07557-3)
Supplement: Supplementary file 4 — Reporting Summary [file 42003_2025_7557_MOESM4_ESM.pdf]

nature portfolio

Corresponding author(s): Lewis Fry, Robert MacLaren

Last updated by author(s): Dec 10, 2024

Reporting Summary

Nature Portfolio wishes to improve the reproducibility of the work that we publish. This form provides structure for consistency and transparency in reporting. For further information on Nature Portfolio policies, see our Editorial Policies and the Editorial Policy Checklist.

Statistics

For all statistical analyses, confirm that the following items are present in the figure legend, table legend, main text, or Methods section.

n/a	Confirmed	
☐	☒	The exact sample size (*n*) for each experimental group/condition, given as a discrete number and unit of measurement
☐	☒	A statement on whether measurements were taken from distinct samples or whether the same sample was measured repeatedly
☐	☒	The statistical test(s) used AND whether they are one- or two-sided<br>*Only common tests should be described solely by name; describe more complex techniques in the Methods section.*
☐	☒	A description of all covariates tested
☐	☒	A description of any assumptions or corrections, such as tests of normality and adjustment for multiple comparisons
☐	☒	A full description of the statistical parameters including central tendency (e.g. means) or other basic estimates (e.g. regression coefficient) AND variation (e.g. standard deviation) or associated estimates of uncertainty (e.g. confidence intervals)
☐	☒	For null hypothesis testing, the test statistic (e.g. F, t, r) with confidence intervals, effect sizes, degrees of freedom and P value noted<br>*Give P values as exact values whenever suitable.*
☒	☐	For Bayesian analysis, information on the choice of priors and Markov chain Monte Carlo settings
☒	☐	For hierarchical and complex designs, identification of the appropriate level for tests and full reporting of outcomes
☒	☐	Estimates of effect sizes (e.g. Cohen's *d*, Pearson's *r*), indicating how they were calculated

Our web collection on statistics for biologists contains articles on many of the points above.

Software and code

Policy information about availability of computer code

Data collection	ABR data collection with BioSigRZ software (Tucker Davis Technologies), ERG data collection using a stimulus protocol detailed in Supp. Table 2 in Espion v6 software (Diagnosys), OCT imaging with HEYEX Software (Heidelberg Engineering)
Data analysis	GraphPad Prism v10 for data analysis and statistics, MultiEditR Software (Kluesner et al. 2021) and CRISPResso2 (v2.1.3) for sequencing analysis of Sanger and NGS data, R (v4.2.0) fisher.test function.

For manuscripts utilizing custom algorithms or software that are central to the research but not yet described in published literature, software must be made available to editors and reviewers. We strongly encourage code deposition in a community repository (e.g. GitHub). See the Nature Portfolio guidelines for submitting code & software for further information.

Data

Policy information about availability of data

All manuscripts must include a data availability statement. This statement should provide the following information, where applicable:

- Accession codes, unique identifiers, or web links for publicly available datasets
- A description of any restrictions on data availability
- For clinical datasets or third party data, please ensure that the statement adheres to our policy

Numerical source data for graphs and plots in the main figures can be found in Supplementary Data 1. Additional data supporting the findings of this manuscript are available upon request.

Research involving human participants, their data, or biological material

Policy information about studies with human participants or human data. See also policy information about sex, gender (identity/presentation), and sexual orientation and race, ethnicity and racism.

Reporting on sex and gender	N/A
Reporting on race, ethnicity, or other socially relevant groupings	N/A
Population characteristics	N/A
Recruitment	N/A
Ethics oversight	N/A

Note that full information on the approval of the study protocol must also be provided in the manuscript.

Field-specific reporting

Please select the one below that is the best fit for your research. If you are not sure, read the appropriate sections before making your selection.

☒ Life sciences ☐ Behavioural & social sciences ☐ Ecological, evolutionary & environmental sciences

For a reference copy of the document with all sections, see nature.com/documents/nr-reporting-summary-flat.pdf

Life sciences study design

All studies must disclose on these points even when the disclosure is negative.

Sample size	Sample sizes used depended on individual experiments depending on variation and expected effect sizes, and determined by common practices in molecular biology. Group sizes are described for each figure. P values of <0.05 were used for statistical significance
Data exclusions	All data was included, except in the case of mice that had complications during subretinal injection preventing the creation of a successful bleb such as haemorrhage, extraocular reflux of injected substance, or inadvertent retinotomy were recorded at the time of surgery, and affected eyes were excluded from analysis
Replication	Experimental replicates were performed for all findings as described in the text
Randomization	Animals were randomised as individual subjects within cages
Blinding	Authors were blinded to group allocation during measurement/acquisition of data, with numbers assigned to experimental subjects to prevent bias during data acquisition

Reporting for specific materials, systems and methods

We require information from authors about some types of materials, experimental systems and methods used in many studies. Here, indicate whether each material, system or method listed is relevant to your study. If you are not sure if a list item applies to your research, read the appropriate section before selecting a response.

Materials & experimental systems

n/a	Involved in the study	
☐	☒	Antibodies
☐	☒	Eukaryotic cell lines
☒	☐	Palaeontology and archaeology
☐	☒	Animals and other organisms
☒	☐	Clinical data
☒	☐	Dual use research of concern
☒	☐	Plants

Methods

n/a	Involved in the study	
☒	☐	ChIP-seq
☒	☐	Flow cytometry
☒	☐	MRI-based neuroimaging

Antibodies

Antibodies used	All antibodies used with reference numbers are available in supplementary table 3
Validation	Commercially available antibodies with validation data available on the suppliers website. The USH2A antibody has previously been validated in multiple publications by Dr Jun Yang.

Eukaryotic cell lines

Policy information about cell lines and Sex and Gender in Research

Cell line source(s)	HEK293T cells (ATCC)
Authentication	Cell lines were not authenticated
Mycoplasma contamination	Not tested
Commonly misidentified lines (See ICLAC register)	Not applicable

Animals and other research organisms

Policy information about studies involving animals; ARRIVE guidelines recommended for reporting animal research, and Sex and Gender in Research

Laboratory animals	All mice were bred on a C57BL/6J background with a repaired Cdh23 allele (Cdh23753A>G), generated by the Mary Lyon Centre MRC Harwell, Oxfordshire UK (See Mianné, J. et al. Genome Med. 8, 16 (2016)). Ush2a -W3947X mice were generated for this study as described in the paper.
Wild animals	NA
Reporting on sex	Sex was equally distributed by randomisation between experimental groups.
Field-collected samples	NA
Ethics oversight	All animal work was performed in accordance with the Animals (Scientific Procedures) Act 1986, UK, with the Association for Research in Vision & Ophthalmology (ARVO) statements on the care and use of animals in ophthalmic research. All procedures were evaluated and approved by the local Animal Research Ethics Committee of the University of Oxford, carried out under a Home Office Project Licence.

Note that full information on the approval of the study protocol must also be provided in the manuscript.

Plants

Seed stocks	NA
Novel plant genotypes	NA
Authentication	NA
